# Vitamin E synthesis and response in plants

**DOI:** 10.3389/fpls.2022.994058

**Published:** 2022-09-14

**Authors:** Yue Niu, Qian Zhang, Jiaojiao Wang, Yanjie Li, Xinhua Wang, Yan Bao

**Affiliations:** ^1^Shanghai Collaborative Innovation Center of Agri-Seeds, Joint Center for Single Cell Biology, School of Agriculture and Biology, Shanghai Jiao Tong University, Shanghai, China; ^2^School of Agriculture and Biology, Shanghai Jiao Tong University, Shanghai, China

**Keywords:** vitamin E, VTE, tocopherol, tocochromanol, biosynthesis, pathway, stress, signal

## Abstract

Vitamin E, also known as tocochromanol, is a lipid-soluble antioxidant that can only be produced by photosynthetic organisms in nature. Vitamin E is not only essential in human diets, but also required for plant environment adaptions. To synthesize vitamin E, specific prenyl groups needs to be incorporated with homogentisate as the first step of reaction. After decades of studies, an almost complete roadmap has been revealed for tocochromanol biosynthesis pathway. However, chlorophyll-derived prenyl precursors for synthesizing tocochromanols are still a mystery. In recent years, by employing forward genetic screening and genome-wide-association approaches, significant achievements were acquired in studying vitamin E. In this review, by summarizing the recent progresses in vitamin E, we provide to date the most updated whole view of vitamin E biosynthesis pathway. Also, we discussed about the role of vitamin E in plants stress response and its potential as signaling molecules.

## Introduction

Vitamin E (also called tocochromanols) is an essential micronutrient for humans, which is produced by phototrophs such as plants, and some algae (Falk and Munné-Bosch, 2010). As antioxidant, vitamin E can convert free radicals into less reactive compounds, playing a pivotal role in human health (Zingg, 2007). Insufficient consumption of vitamin E could cause many diseases, including cancers, Alzheimer’s disease and cardiovascular disease, but not limited (Sen et al., 2007; Falk and Munné-Bosch, 2010; Sozen et al., 2019).

**FIGURE 1:**
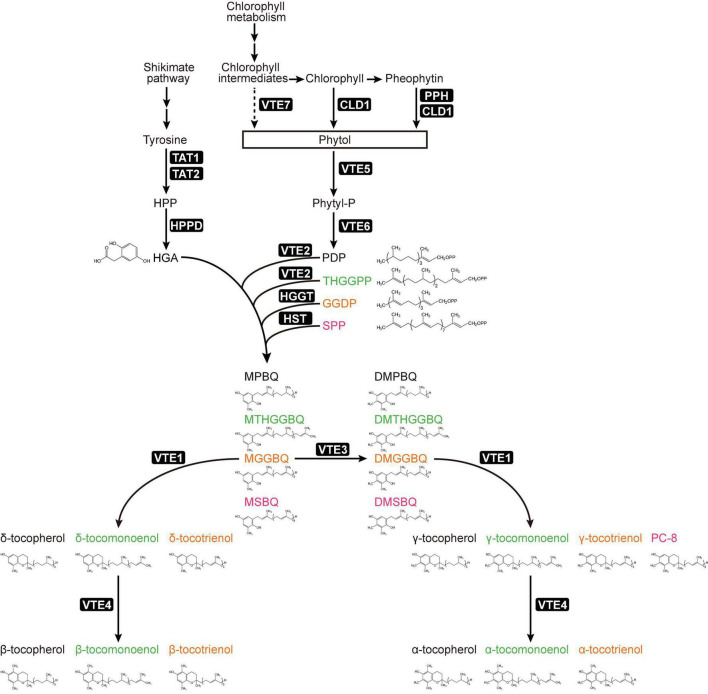
Plant tocochromanol biosynthesis pathway and sources of metabolites. Metabolite names were colored for recognizing different tocochromanol synthesis pathways. Abbreviations of metabolites: HPP, 4-hydroxyphenylpyruvate; HGA, homogentisate; Phytyl-P, phytyl-phosphate; PDP, phytyl-diphosphate; THGGPP, tetrahydrogeranylgeranyl pyrophosphate; GGDP, geranylgeranyl pyrophosphate; SPP, solanesyl pyrophosphate; MPBQ, 2-methyl-6-phytyl-1,4-benzoquinol; DMPBQ, 2,3-dimethyl-6-phytyl-1,4-benzoquinol. Abbreviations of enzymes: TAT1, tyrosine aminotransferase 1; TAT2, tyrosine aminotransferase 2; HPPD, 4-hydroxyphenylpyruvate dioxygenase; CLD1, chlorophyll dephytylase 1; PPH, pheophytin pheophorbide hydrolase; VTE, vitamin E biosynthetic enzyme.

Tocochromanol molecule contains a polar chromanol ring head and a prenyl side chain. According to the various types of side chains, tocochromanols can be defined as tocopherol, tocotrienol, plastochromanol-8 (PC-8) and tocomonoenol (Figure 1). Tocopherol contains fully saturated aliphatic side chain, while the side chain of tocotrienol contains three extra trans double bonds. PC-8 has similar unsaturated as tocotrienol but longer side chain, whereas tocomonoenol has only one double bond on its side chain (Tian et al., 2007; Sadre et al., 2010; Stacey et al., 2016). Based on the differences in numbers and positions of methyl groups on the chromanol ring head, tocochromanol isoforms can be classified as α, β, γ, and δ (Figure 1). While for PC-8, only δ-form was found in nature (Kamal-Eldin and Appelqvist, 1996).

Tocopherols are ubiquitously synthesized in all plant species and especially abundant in photosynthetic tissues and seeds. Tocotrienols are mostly concentrated in monocot species like maize (*Zea mays*) embryo (Falk and Munné-Bosch, 2010), and the three extra double bonds in tocotrienols were believed to able to confer greater potential for scavenging peroxyl radicals (Suzuki et al., 1993). PC-8 was first identified in the leaves of the rubber tree and later found to be also enriched in *Brassica napus*, tomato fruit, and tuber of *Dioscorea alata* (Goffman and Mollers, 2000; Cheng et al., 2007; Zbierzak et al., 2010), and the concentrations of leaf PC-8 are differed by species and developmental stages (Whittle et al., 1965; Kruk et al., 2014). Studies of tocomonoenols suggested that they were mainly accumulated in the seed oil from palm, Slovenia pumpkin and sunflower (Matsumoto et al., 1995; Butinar et al., 2011; Hammann et al., 2015), but the exact function of tocomonoenols *in planta* are yet to be thoroughly verified.

In the past few decades, *Arabidopsis thaliana* has been employed as a plant model for dissecting tocopherol function and its biosynthesis pathway, and many key VTE (*V*i*T*amin *E*) enzymes were identified. Recently, by applying EMS-based forward genetic screening and genome-wide association study (GWAS), several key chlorophyll metabolic enzymes were identified to be required for tocopherol biosynthesis, including POR, CLD1 and VTE7 etc. (Lin et al., 2016; Diepenbrock et al., 2017; Wang et al., 2018; Hershberger et al., 2022; Wu et al., 2022). In this review, we summarize the recent progresses in tocochromanol biosynthesis, discussing the role of tocochromanol in plant stress response, and its potentials in signal transduction.

## Biosynthesis of vitamin E

To produce tocochromanols, homogentisate (HGA) will be condensed with different prenyl chains by homogentisate phytyltransferase [HPT, also named VTE2 (*V*i*T*amin *E* 2 loci)], in one-to-one ratio (Figure 1). HPT genes can be found in all green plants, and some algae including cyanobacterium *Synechocystis* (Collakova and DellaPenna, 2001; Savidge et al., 2002). For tocopherols, HGA and phytyl diphosphate (PDP) are catalyzed by VTE2 to generate 2-methyl-6-phytyl-1,4-benzoquinol (MPBQ). VTE2 can also use the tetrahydrogeranylgeranyl pyrophosphate (THGGPP) to generate 2-methyl-6-tetrahydrogeranylgeranyl-1,4-benzoquinol (MTHGGBQ) for producing tocomonoenol in Arabidopsis seeds (Pellaud et al., 2018). In monocots, homogentisate geranylgeranyl transferases (HGGTs) are seed-specific and plastid-targeted, which can condense geranylgeranyl pyrophosphate (GGDP) instead of PDP with HGA to generate 2-methyl-6-geranylgeranyl-1,4-benzoquinol (MGGBQ) for tocotrienol biosynthesis (Cahoon et al., 2003; Yang et al., 2011). Although HGGT and VTE2 are close in structure, their enzyme activities toward different substrates vary quite much. For example, the activity of barley HPPT toward GGDP is 6 times higher than that of PDP; the VTE2 enzyme confers 9 times higher activity toward PDP than that of GGDP. Interestingly, barley HPPT can restore the levels of both tocopherols and tocotrienols in Arabidopsis *vte2* mutant (Yang et al., 2011). Moreover, homogentisate solanesyltransferase (HST) can condense HGA and solanesyl pyrophosphate (SPP) to produce the 2-methyl-6-solanesyl-1,4-benzoquinol (MSBQ), the precursor of PC-8 (Sadre et al., 2006; Tian et al., 2007).

The downstream of vitamin E biosynthesis pathway is divided into two branches. In one branch, the MPBQ, MGGBQ, MSBQ, and MTHGGBQ are methylated by a methyltransferase (MT, VTE3) to produce DMPBQ, DMGGBQ, DMTHGGBQ, and DMSBQ (PQ-9), respectively, the precursors of α- and γ-tocochromanols (Cheng et al., 2003; Van Eenennaam et al., 2003). Then the methylated compounds are cyclized by tocopherol cyclase (TC, VTE1) to produce γ-tocochromanols (Cheng et al., 2003). In the other branch of this pathway, the demethylated compounds (MPBQ, MGGBQ, MSBQ, and MTHGGBQ) are cyclized directly by VTE1 to produce δ-tocochromanols (Cheng et al., 2003). In the final step, the γ- and δ-tocochromanols, respectively, are methylated to produce α- and β-tocochromanols by γ-tocopherol methyltransferase (γ-TMT, VTE4) (Shintani and DellaPenna, 1998; Bergmüller et al., 2003).

Biosynthesis of tocochromanols is mainly carried out in the plastid, but one of its main precursors HGA, which provides the chromanol ring head for tocochromanols, is produced during L-tyrosine (Tyr) degradation in the cytoplasm (Figure 1). Through the shikimate pathway, tyrosine aminotransferases (TATs) catalyze reversible reaction between Tyr and 4-hydroxyphenylpyruvate (HPP), and at least two homologous genes *TAT1* and *TAT2* were identified in the genome of *Arabidopsis thaliana* (Siehl et al., 2014; Stacey et al., 2016; Wang et al., 2016). Arabidopsis *TAT1* gene loss of function mutant showed reduced tocopherols under normal condition (Riewe et al., 2012), but the *tat2* single mutants have no effect on Tyr and tocopherol levels. In the *tat1 tat2* double mutants, compared with wild-type and *tat* single mutants, more Tyr were accumulated and fewer tocopherols were maintained, and this effect was amplified under high-light stress (Wang et al., 2019). TAT1 and TAT2 thus work redundantly in Tyr degradation and tocopherol biosynthesis, with TAT1 playing a major role. Then, HPP is converted to HGA by the 4-hydroxyphenylpyruvate dioxygenase (HPPD), which is encoded by a single-copy gene in Arabidopsis (Tsegaye et al., 2002). However, HPPDs possess different subcellular localizations according to studies in various plant species (Pellaud and Mène-Saffrané, 2017). For example, HPPD proteins of *Spinacia oleracea*, *Lemna gibba* and maize are targeted to plastids, while in carrot and Arabidopsis they are located in the cytoplasm (Löffelhardt and Kindl, 1979; Fiedler et al., 1982; Garcia et al., 1997; Siehl et al., 2014; Wang et al., 2016).

Another main precursor of tocopherols comes from phytyl diphosphate (PDP), which is generated from two steps of phosphorylation relay using phytol (Gutbrod et al., 2019). The first step is to generate phytyl-phosphate via VTE5 (phytol kinase), which accounts for 80% and 50% total tocopherol biosynthesis in Arabidopsis seeds and leaves, respectively (Valentin et al., 2006). The second step is executed by VTE6 (phytyl phosphate kinase), and due to over accumulation of phytyl-phosphate, *vte6* mutant plant confers severe growth defects. By introducing *vte5* into the *vte6* background, the *vte5 vte6* double mutant resembles wild-type in plant growth, but showing tocopherol deficient and high levels of accumulated chlorophylls (Vom Dorp et al., 2015). GGDP, SPP and THGGPP are the polyprenyl chain precursors of tocotrienols, PC-8 and tocomonoenols, respectively (Mène-Saffrané, 2018). Of note, all of the four tocochromanol isoprenoid side chains can be produced by GGDP synthases (Pellaud and Mène-Saffrané, 2017).

However, accumulated studies have suggested that the phytol group used for tocopherol biosynthesis mostly comes from the chlorophyll degradation (Valentin et al., 2006; Gutbrod et al., 2019). Thus, finding the relevant hydrolases that can bring down phytol group from chlorophyll and/or chlorophyll derivatives is key for dissecting tocopherol biosynthesis pathway. The first chlorophyllase (CLH) was isolated from *Citrus sinensis*, which shows activity toward chlorophylls, and two *CLH* genes (*AtCLH1* and *AtCLH2*) were found in Arabidopsis (Jacob-Wilk et al., 1999; Tsuchiya et al., 1999). The highest transcripts of *AtCLH1* and *AtCLH2* were found in young leaves and their levels decline gradually during leaf maturation (Chen et al., 2014; Tian et al., 2021). However, previous studies had shown that the two Arabidopsis CLHs were not required for chlorophyll breakdown during leaf senescence. In addition, CLH proteins are targeted to vacuole instead of chloroplasts. Based on functional genomic approach with the features of senescence-related regulation and chloroplast targeting, Schelbert et al. identified a hydrolase called pheophytin pheophorbide hydrolase (PPH) in Arabidopsis. The *pph* mutant showed stay-green phenotype during senescence, compared with its wild-type control. *In vitro*, PPH confers specific activity toward pheophytin but not chlorophylls (Schelbert et al., 2009). Recently, through EMS screening for Arabidopsis heat-sensitive progenies, an Arabidopsis *CHLOROPHYLL DEPHYTYLASE1* (*CLD1*) gene was identified. A G-to-A transition at position 957 of *cld1* gene results in the replacement of Gly-193 by Asp (G193D), which confers much higher activity of *cld1* toward both chlorophylls and pheophytin than that of its native CLD1 (Lin et al., 2016). Following studies found about 15% tocopherol increase in CLD1 and *cld1* overexpression plants, but no significant difference in its miRNA lines (Lin and Charng, 2017). Although CLHs, PPH and CLD1 can cleave phytol directly from chlorophyll and/or pheophytin, none of the single or high order mutants (*clh1/clh2*, *pph*, and *pph/clh1/clh2*) significantly affect tocopherol contents. Moreover, overexpression of the four genes only moderately increased the levels of tocopherol, suggesting the relevant alpha/beta hydrolase is yet to be identified (Zhang et al., 2014; Lin and Charng, 2017). Recently, GWAS of seed tocopherols was applied using 814 Arabidopsis natural variation lines (part of the 1001 Arabidopsis Genome Panel, Alonso-Blanco et al., 2016), a novel seed-specific alpha/beta hydrolase gene *AtVTE7* was identified. AtVTE7 is targeted to the chloroplast envelope and accounts for 55% of total seed tocopherols. Consistent with the results in Arabidopsis, the maize orthologous gene *ZmVTE7* controls 38% and 49% total tocopherols in kernel and leaf, respectively (Albert et al., 2022). Of note, Arabidopsis AtVTE7 is only detected in seed, does not affect tocopherol trait in leaf. Although VTE7 can provide phytol from chlorophyll degradation for tocopherol biosynthesis, this enzyme mainly affects the levels of chlorophyll biosynthetic intermediates, instead of bulk chlorophyll levels (Albert et al., 2022).

In addition to the alpha/beta hydrolases that are directly involved in phytol production, many chlorophyll-metabolism-related genes also contribute to tocopherol homeostasis. For instance, plant NYE [Non-Yellowing, also named SGR (Stay-Green)] are chloroplast-localized Mg-dechelatase proteins, and during chlorophyll degradation NYEs closely cooperate with other chlorophyll catabolic enzymes including PPH (Armstead et al., 2007; Sato et al., 2007; Zhang et al., 2014). Indeed, the seed tocopherol levels of the Arabidopsis double mutant *nye1 nye2* modestly reduced, compared with that in the wild-type (Zhang et al., 2014). Chlorophyll synthesis needs the esterizing of chlorophyllide with either GGDP or PDP. The RNAi lines of *CHLOROPHYLL SYNTHASE* (*CHLSYN*) exhibit significantly reduced chlorophylls but up to 2 times increased tocopherols (Zhang et al., 2015). Moreover, two QTLs that encode homologs of protochlorophyllide reductase (*POR1* and *2*) were revealed in maize, and the two *por* loci had the highest phenotypic variance explained for all four forms of tocopherols calculated (Diepenbrock et al., 2017; Wang et al., 2018). Thus, disturbing the genes involved chlorophyll metabolism can affect tocopherol biosynthesis, suggesting a precise mechanism for balancing chlorophyll metabolism and tocopherol biosynthesis in plants.

## Vitamin E in stress response

**FIGURE 2:**
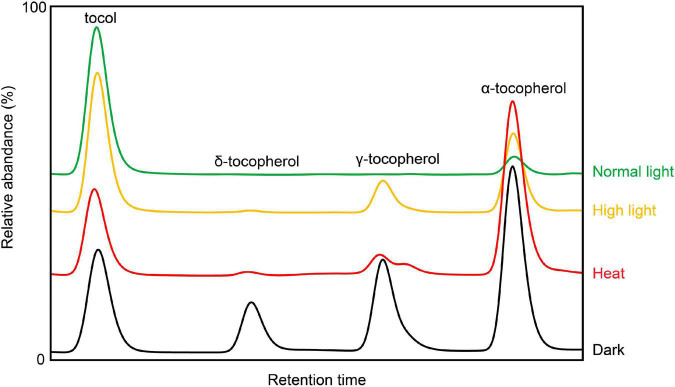
Representative HPLC traces of Arabidopsis leaf tocopherols subjected to different growth conditions (high light, heat, and dark). Tocol is used as internal standard [based on Bao et al. (2020), with modification].

**FIGURE 3:**
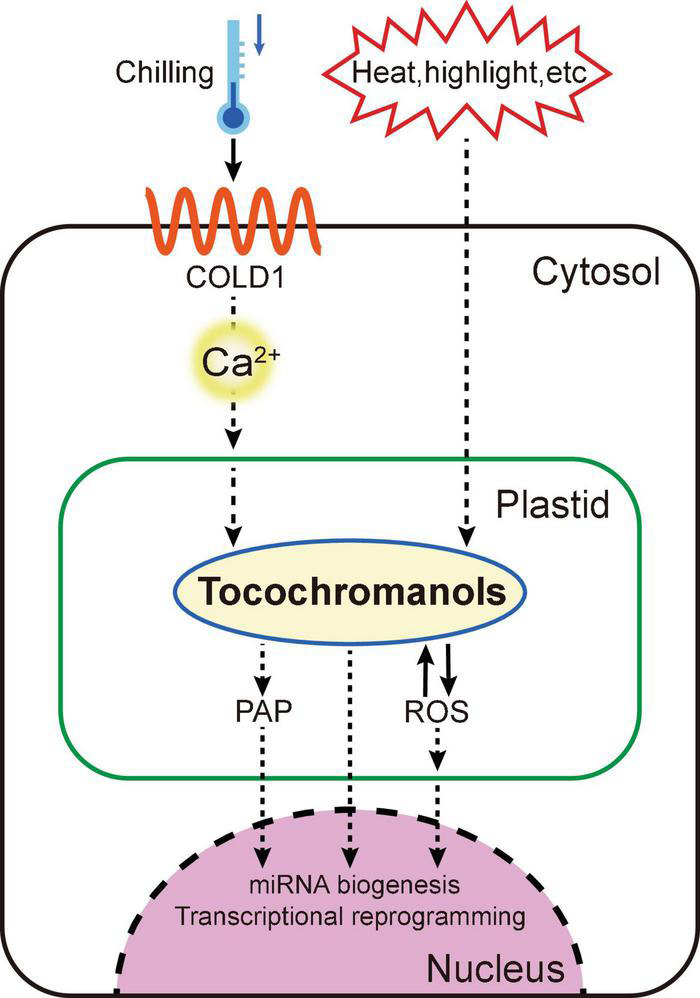
A proposed working model of the vitamin E in signal transduction Chilling stress can be sensed by COLD1 to regulate calcium signal and tocochromanol homeostasis for transcriptional reprogramming in the nucleus. Other stresses including high-light and heat can also transduce retrograde signaling between chloroplast and nucleus for miRNA biogenesis, via manipulating PAP (3′-phosphoadenosine-5′-phosphate) and tocochromanols.

As important antioxidants, tocochromanols can be boosted to high levels during various biotic and abiotic stresses (Figures 2, 3) (Bao et al., 2020). Meanwhile, plants with low levels of tocochromanols are more susceptible to different stressful treatments, suggesting a crucial role of vitamin E in plant environment adaptions.

Temperature and light intensity are the two key environmental factors that can affect crop yield (Pretty et al., 2010). During high light and heat stress, tocopherols (especially α-tocopherol) are induced to accumulate at high levels, and elevated α-tocopherols were believed to be required for protecting photosystem from savaging singlet oxygen and maintaining the stability of chloroplast (Kruk et al., 2005). Indeed, tocopherol deficient Arabidopsis mutants are more vulnerable to high light (Kobayashi and DellaPenna, 2008). When grown under low temperature, vitamin E deficient mutants are retarded in plant growth (Maeda et al., 2006), which mainly attributes to defects in phloem loading, coincide with the findings in maize and potato (*Solanum tuberosum*) (Russin et al., 1996; Hofius et al., 2004). The combination of high light and low temperature causes strong lipid peroxidation and photooxidative stresses to Arabidopsis, and this effect was exemplified in *vte1* mutant (Havaux et al., 2005). In maize, compared with high temperature, more tocopherols and tocotrienols were produced under low temperature (Xiang et al., 2019). In addition, tomato *SlVTE5* silenced plants display a strong chlorotic phenotype with low levels of α-tocopherol under the stress combined with high-light and high-temperature (Spicher et al., 2017). Moreover, high-light stress also triggers the expression of *HPPDs* in *Medicago sativa* and *Lactuca sativa* for counteracting and survival strategies (Ren et al., 2011; Jiang et al., 2017).

Drought is one of the most common stresses in limiting farming, which leads to significant yield losses (Godwin and Farrona, 2020). The capacity of HPPDs to resist drought has also been demonstrated in various plant species, including *Lactuca sativa*, Medicago and sweet potato (Ren et al., 2011; Jiang et al., 2017; Kim et al., 2021). In rice, *OsVTE1* is induced to significantly high levels under drought stress (Ouyang et al., 2011), and ectopic overexpression of *AtVTE1* in tobacco can enhance tolerance to drought stress via reducing lipid peroxidation, electrolyte leakage and H_2_O_2_ content (Liu et al., 2008). Moreover, overexpression of *MsVTE4* increases the levels of both α-tocopherols and total tocopherols in alfalfa, alleviating oxidative damages, leading to higher tolerance to drought stress (Ma et al., 2020).

Soil with unfavorable high level of soluble salts causes salinity stress in plants, limiting crop yield and the area for farming. In tobacco (*Nicotiana tabacum*) *VTE2* silenced plants, total tocopherols decreased 98%, and ion homeostasis was disturbed with sorbitol and methyl viologen treatment (Abbasi et al., 2007). Meanwhile, in tobacco *VTE4* silenced plants, α- and γ-tocopherols were found to play diversified roles in plant stress tolerance (Abbasi et al., 2007). On the other hand, overexpressing *AtVTE4* can reduce superoxide contents, lipid peroxidation and ion leakage under salt stress (Jin and Daniell, 2014). By employing Arabidopsis tocopherol deficient mutants *vte1* (deficient in α- and γ-tocopherols) and *vte4* (over accumulation of γ-tocopherols), Surówka et al. (2020) revealed that α-tocopherols had stronger regulatory effect than γ-tocopherols through modulating chloroplast biosynthesis pathways and ROS/osmotic-associated compounds under salt stress. Based on the studies above, *AtVTE2* and *AtVTE4* have been engineered in potato breeding for counteracting heavy metal stress (Upadhyaya et al., 2021).

Tocochromanols not only protect plant from abiotic stress, but also contribute to the resistance of biotic stress. *Pseudomonas syringae* can infect a wide range of plant species and leads to serious economic loss (Bohlmann and Keeling, 2008), which is served as a model for dissecting the mechanism of plant-pathogen interactions (Xin et al., 2018). Arabidopsis *vte2* mutants exhibit stronger lipid peroxidation and produce less SA (salicylic acid, a key phytohormone in transducing pathogen signal), resulting in susceptibility to *P. syringae* (Stahl et al., 2019). As a necrotrophic fungus, *Botrytis cinereal* infects many important plant species, including Arabidopsis (Staats et al., 2005). Through detailed analysis and comparison of lipid and hormone changes in *B. cinereal-*infected Arabidopsis *vte1* and *vte4* mutants, Cela et al. found that altered tocopherol compositions could reduce plant tolerance and delay the activation of defense pathway (Cela et al., 2018).

## Vitamin E in signal transduction

Vitamin E has long been assessed and studied as an antioxidant, but emerging evidences strongly suggested that vitamin E may also serve as signaling molecules in plants. Reactive oxygen species (ROS) such hydroperoxide and single oxygen that produced in the photosystem have been shown as important signals in the communications between chloroplast and gene expression in the nucleus (Figure 3) (Foyer and Noctor, 2003, 2005). When single oxygen accumulated in the chloroplast, tocopherols will be oxidized to produce tocopheryl-radical and hydroperoxide. Reversibly, these two products can be reduced to tocopherols by introducing ascorbate (also known as vitamin C) (Neely et al., 1988). Thus, through eliminating ROS, vitamin E maintains chloroplast redox state and modulates retrograde signaling from the chloroplast to nucleus (Figure 3) (Krieger-Liszkay and Trebst, 2006).

Cross-talks between vitamin E and phytohormones were also revealed. For instance, tocopherol deficient *vte1* mutants accumulate more jasmonic acid (JA) and anthocyanin than that of wide-type, causing growth retardation in both high-light and low temperature conditions (Munné-Bosch et al., 2007). During low phosphate (Pi) treatment, both *vte1* and *vte4* mutants accumulate more JA and SA than that in the wide-type. In addition, the expression levels of over 500 transcription factors are significantly affected in *vte1* and *vte4* plants, indicating that tocopherols are involved in phytohormone signaling and transcriptional reprogramming (Allu et al., 2017). In another case, ethylene-responsive *cis* elements were found in the promoter region of Mango (*Mangifera indica*) *MiHPPD* gene. During fruit ripening and leaf senescence, elevated endogenous ethylene can induce *MiHPPD* expression, giving rise to tocopherols contents (Singh et al., 2011). Overall, interactions and communications between vitamin E and phytohormones contribute to plant environment adaptations via fine-tuning downstream gene expressions.

The quantitative trait locus COLD1 was identified recently in japonica rice that can confer tolerance to chilling stress. For sensing low temperature, COLD1 can interact with RGA1 (G-protein α subunit) to activate the Ca^2+^ channel and accelerate GTPase activity of G-protein. Influx of calcium, an important intracellular second messenger, activates gene transcription of vitamin E and vitamin K1 biosynthesis pathways, promoting plant cold tolerance (Luo et al., 2021). Another tocopherol-mediated chloroplast-to-nucleus signaling event was discovered during the study of heat stress-associated microRNA (miRNA) biogenesis. The metabolite 3′-phosphoadenosine-5′-phosphate (PAP) has been broadly racialized as crucial secondary messenger in plant stress responding. Heat stress promotes accumulation of tocopherols, and in turn produces more PAP, which inhibits the nuclear exoribonucleases (XRN), stimulating the biogenesis of microRNAs including miR398 to enhance plant heat tolerance (Fang et al., 2019).

Studies in human and animals suggested that tocopherol-binding protein (TBP) is important for the distribution and transport of α-tocopherol among different tissues. In the latest research, Bermúdez et al. identified the SlTBP (*Solanum lycoperisicum* tocopherol-binding protein) as a homolog of the human α-tocopherol transfer protein (HsTTP). *In vitro* biochemical assay suggested that SlTBP possesses α-tocopherol binding ability. SlTBP is chloroplast-targeted, and knocking down *SlTBP* expression in tomato confers disorders in tocopherol, carotenoid and lipid compositions. Finding of TBP in plants sheds light on understanding vitamin E transport, implying its potential as signaling molecules (Bermúdez et al., 2018).

## Future perspectives

Studies in Arabidopsis indicated that 90% and more of the total tocopherols (∼5 ng/mg fresh weight) in leaves are α-tocopherol, while in Arabidopsis seeds, tocopherols (∼370 ng/mg dry seed) are dominated by the γ isoform. Crucial physiological functions of vitamin E for plants were exemplified by the observation that tocopherol defective Arabidopsis mutants were severely affected in seed longevity, germination and seeding growth (Sattler et al., 2004). Differential regulation of the same cassette of genes for tocochromanol biosynthesis in different tissues warrants for future explorations. Chlorophyll and its relevant derivatives are the most abundant pigments in green plants, and accumulated evidences suggested that chlorophyll-derived phytol groups are the main source for vitamin E biosynthesis (Gutbrod et al., 2019). VTE7 is a novel alpha/beta hydrolase that fits in the missing gap between chlorophyll metabolism and vitamin E production, accounting for more than 50% of total tocopherol biosynthesis in seeds. However, its exact targets still need to be verified. In recent years, tocopherols were found to be involved in both cold response and PAP-mediated retrograde signal transduction. In addition, tocopherol binding protein (TBP) was identified in tomato. Thus, role of tocopherols in acting as signaling transducers are promising and deserved to be investigated in depth. More importantly, as an essential nutrient, engineering balanced vitamin E in crops like soybean, rapeseed will advance plant breeding and benefit human health.

## Author contributions

YB concepted the topic of this manuscript and revised the manuscript. YN and QZ drafted the manuscript with YB. All authors contributed to the article and approved the submitted version.
